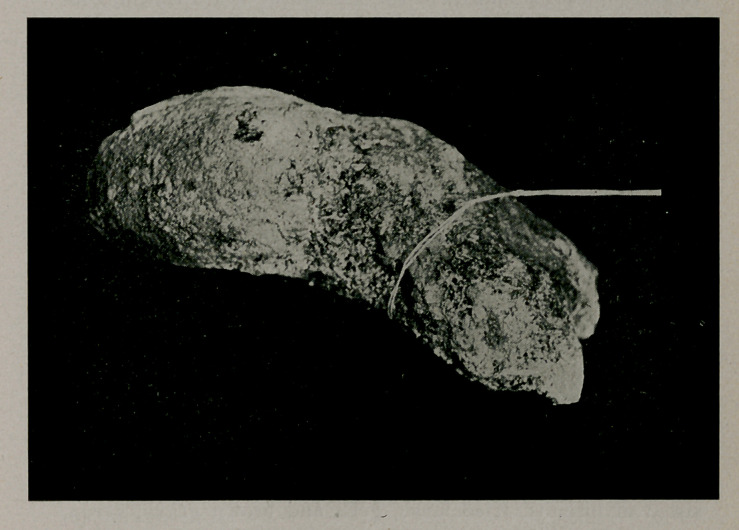# Largest Urethral Calculus in the World

**Published:** 1915-12

**Authors:** 


**Largest Urethral Calculus in the World.** Dr. S. E. Earp, editor of the Indianapolis Medical Journal, kindly allows us to reproduce the accompanying cuts. The calculus was removed from a woman aged 47, weighs 845½ grains, is 3 inches long, attains a diameter of 1 1-8 inches and a circumference of 4 1-8 inches. It is phosphatic and extended into the bladder, causing an ulceration, The case was first reported in the N. Y. Med. Jour., Feb. 2, 1907.

## Figures and Tables

**Figure f1:**
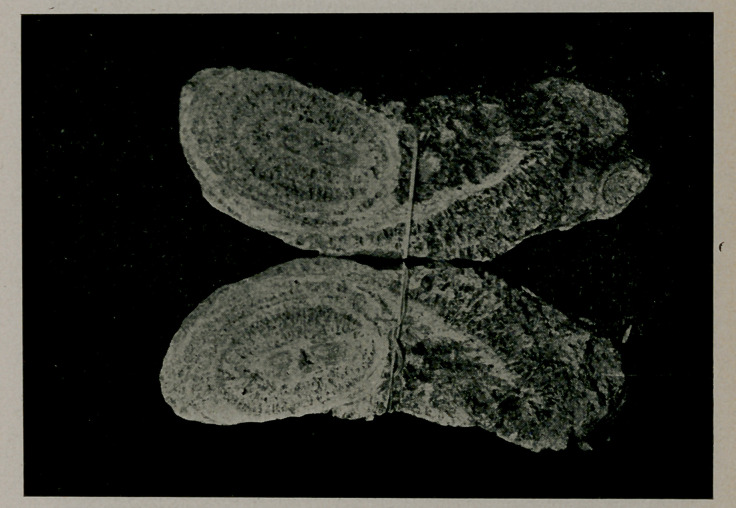


**Figure f2:**